# Supplementary material for: Monitoring Human Milk β-Casein Phosphorylation and O-Glycosylation Over Lactation Reveals Distinct Differences between the Proteome and Endogenous Peptidome
Source: Int J Mol Sci. 2021 Jul 29;22(15):8140. doi: 10.3390/ijms22158140 (PMC8347866; doi:10.3390/ijms22158140)
Supplement: Supplementary file 1 [file ijms-22-08140-s001.zip › ijms-1257229-supplementary.pdf]

Supplemental Table S1: Glycoproteoforms detected between both donors across lactation for both low and high resolutions LC-MS experiments

**Detected proteoform**	**Theoretical mass (kDa)**		**Experimental mass (kDa; average ± stdev)**		**Retention time apex (min; average ± stdev)**
	**Monoisotopic**	**Average**	**Monoisotopic**	**Average**	
**β-casein 0P**	23842.82	23857.50	23843.05 ± 0.02	23857.36 ± 0.19	16.71 ± 0.11
**β-casein 1P**	23922.79	23937.48	23923.00 ± 0.20	23937.27 ± 0.16	16.83 ± 0.12
**β-casein 2P**	24002.75	24017.46	24002.97 ± 0.19	24017.28 ± 0.10	17.06 ± 0.07
**β-casein 3P**	24082.72	24097.44	24082.94 ± 0.02	24097.25 ± 0.09	17.28 ± 0.08
**β-casein 4P**	24162.69	24177.42	24162.90 ± 0.01	24177.27 ± 0.09	17.51 ± 0.06
**β-casein 5P**	24242.65	24257.40	24242.88 ± 0.01	24257.22 ± 0.08	17.81 ± 0.05
**β-casein 0P N_1**	24045.90	24060.69	24047.00 ± 0.03	24062.20 ± 0.29	17.12 ± 0.09
**β-casein 0P N_1H_1**	24207.95	24222.84	24208.25 ± 0.47	24223.32 ± 0.62	17.51 ± 0.19
**β-casein 1P N_1**	24125.87	24140.67	24126.42 ± 0.73	not detected	17.33 ± 0.05
**β-casein 1P N_1H_1**	24287.92	24302.82	24288.31 ± 0.50	24302.97 ± 0.57	17.72 ± 0.42
**β-casein 2P N_1**	24205.83	24220.65	24205.03 ± 0.34	not detected	17.53 ± 0.08
**β-casein 2P N_1H_1**	24367.89	24382.80	24368.69 ± 0.60	24383.22 ± 0.45	16.57 ± 0.14
**β-casein 3P N_1**	24285.80	24300.63	24284.89 ± 0.01	not detected	17.88 ± 0.00
**β-casein 3P N_1H_1**	24447.85	24462.78	not detected	24464.17 ± 0.01	16.52 ± 0.21
**β-casein 4P N_1H_1**	24527.82	24542.76	not detected	24542.32 ± 0.73	17.01 ± 0.12
**β-casein 4P N_2H2**	24892.95	24908.09	not detected	24907.34 ± 0.08	16.96 ± 0.00

*P is phosphorylation, H = hexose, N = *N*-acetylhexosamine

Supplemental Table S2: Curation criteria used to determine proper score settings reducing the FDR

**Filtering conditions**	**Proteome derived data**				**Peptidome derived data**			
	**NM* PSMs**	**Glyco PSMs**	**False NM PSMs**	**False Glyco PSMs**	**NM PSMs**	**Glyco PSMs**	**False NM PSMs**	**False Glyco PSMs**
None	73,278	18,488	11,839	6,783	86,174	33,747	6,701	5,864
**Percent of total**	**25**		**16**	**37**	**39**		**8**	**17**
First AA > 15	71,747	17,285	10,943	6,274	79,612	27,235	6,230	5,448
**Percent of total**	**24**		**15**	**36**	**34**		**8**	**20**
Score ≥ 150	50,047	61,89	310	26	58,367	10,444	87	32
**Percent of total**	**12**		**0.6**	**0.4**	**18**		**0.1**	**0.3**
Score + log probability ≥ 1.5	49,091	5,895	100	0	57,490	99,50	14	7
**Percent of total**	**12**		**0.2**	**-**	**17**		**0.02**	**0.07**
Score + log probability + Delta modification ≥ 5	44,667	2,286	97	0	48,295	2,241	6	1
**Percent of total**	**5**		**0.2**	**-**	**5**		**0.01**	**0.04**

*Non-modified (NM)

Supplemental Table S3: PSM counts of phospho- and glycosite modifications by donor and time in the peptidome and proteome derived data

Donor one peptidome derived data

	Location	W1	W2	W3	W4	W6	W8	W10	W12	W16	Sum	Average
**Peptidome Phosphorylation**	Thr18	21	42	78	43	49	70	80	50	75	**508**	**56**
	Ser21	405	278	460	319	345	398	429	405	428	**3467**	**385**
	Ser23	521	347	544	381	440	513	581	574	577	**4478**	**498**
	Ser24	607	462	664	526	568	708	783	826	826	**5970**	**663**
	Ser25	610	477	656	512	594	681	788	828	839	**5985**	**665**
	Ser28		4	1	2	4	2	7	4	5	**29**	**4**
	Thr30		1								**1**	**1**
	Thr207	11	8			9	3	1			**32**	**6**
	Thr214	1	5			3					**9**	**3**
	Ser225										**0**	**0**
	***Sum**	**2164**	**1606**	**2402**	**1781**	**1996**	**2370**	**2661**	**2683**	**2745**		
	***Average**	**433**	**321**	**480**	**356**	**399**	**474**	**532**	**537**	**549**		
**Peptidome Glycosylation**	**Location**	**W1**	**W2**	**W3**	**W4**	**W6**	**W8**	**W10**	**W12**	**W16**	**Sum**	**Average**
	Thr18			1			3	1	2	2	**9**	**2**
	Ser21	1		3		4	7	1	4	8	**28**	**4**
	Ser23		1	3	1	2	5	3	6	8	**29**	**4**
	Ser24		1	2	1	1		4	4	7	**20**	**3**
	Ser25			2			1	3	6	8	**20**	**4**
	Ser28	4	1	1	2	1	3	9	5	14	**40**	**4**
	Thr30	5	2	1	2	1	2	8	6	11	**38**	**4**
	Thr207	149	103	20	17	171	23	39	46	51	**619**	**69**
	Thr214	182	166	46	34	217	37	50	57	63	**852**	**95**
	Ser225	31	29	7	5	31	6	5	9	11	**134**	**15**
	***Sum**	**362**	**298**	**73**	**56**	**419**	**66**	**94**	**112**	**125**		
	***Average**	**121**	**99**	**24**	**19**	**140**	**22**	**31**	**37**	**42**		
**Peptidome non-modified**	**Location**	**W1**	**W2**	**W3**	**W4**	**W6**	**W8**	**W10**	**W12**	**W16**	**Sum**	**Average**
	Thr18	583	444	590	486	587	698	765	864	836	**5853**	**650**
	Ser21	215	229	233	244	330	411	476	575	544	**3257**	**362**
	Ser23	106	187	163	205	277	323	364	440	449	**2514**	**279**
	Ser24	25	79	46	64	159	141	171	201	216	**1102**	**122**
	Ser25	26	72	59	89	142	176	180	207	223	**1174**	**130**
	Ser28	639	553	720	612	738	871	974	1052	1067	**7226**	**803**
	Thr30	639	555	724	615	744	875	984	1057	1075	**7268**	**808**
	Thr207	765	733	259	203	721	228	247	286	381	**3823**	**425**
	Thr214	727	653	270	226	660	249	267	318	403	**3773**	**419**
	Ser225	737	688	312	253	709	279	309	342	424	**4053**	**450**
	**Sum**	**6987**	**6124**	**4097**	**3555**	**7139**	**4859**	**5630**	**6319**	**7167**		
	**Average**	**388**	**340**	**228**	**198**	**397**	**270**	**313**	**351**	**398**		

* The Indicated Sum and Average were calculated using on sites that showed evidence of the respective PTM. For phosphosites, only Thr18-Ser25 were considered; for *O*-glycosylation, only sites Thr207-Ser225 were considered. Serine (Ser), Threonine (Thr), weeks (W)

Donor one proteome derived data												
	**Location**	**W1**	**W2**	**W3**	**W4**	**W6**	**W8**	**W10**	**W12**	**W16**	**Sum**	**Average**
**Proteome Phosphorylation**	Thr18				3	1					**4**	**2**
	Ser21	15	3	67	81	6		23	7	12	**214**	**27**
	Ser23	50	51	140	150	47	42	78	76	82	**716**	**80**
	Ser24	103	125	253	287	187	138	221	235	226	**1775**	**197**
	Ser25	93	117	257	279	186	140	222	233	228	**1755**	**195**
	Ser28								1		**1**	**1**
	Thr30										**0**	**0**
	Thr207	1	6	1	5			3	2	1	**19**	**3**
	Thr214		1	1							**2**	**1**
	Ser225		1	1	2		1		2	2	**9**	**2**
	***Sum**	**261**	**296**	**717**	**800**	**427**	**320**	**544**	**551**	**548**		
	***Average**	**261**	**74**	**179**	**160**	**85**	**107**	**136**	**138**	**137**		
**Proteome Glycosylation**	**Location**	**W1**	**W2**	**W3**	**W4**	**W6**	**W8**	**W10**	**W12**	**W16**	**Sum**	**Average**
	Thr18			1							**1**	**1**
	Ser21			2							**2**	**2**
	Ser23			2							**2**	**2**
	Ser24			2	2	1	1	1	2	1	**10**	**1**
	Ser25			1	2	1	1	1	2	1	**9**	**1**
	Ser28			1					1		**2**	**1**
	Thr30			1					1		**2**	**1**
	Thr207	55	66	88	119	74	96	99	105	111	**813**	**90**
	Thr214	100	103	115	148	88	141	125	124	138	**1082**	**120**
	Ser225	9	16	13	24	14	23	14	18	26	**157**	**17**
	***Sum**	**164**	**185**	**216**	**291**	**176**	**260**	**238**	**247**	**275**		
	***Average**	**55**	**62**	**72**	**97**	**59**	**87**	**79**	**82**	**92**		
**Proteome non-modified**	**Location**	**W1**	**W2**	**W3**	**W4**	**W6**	**W8**	**W10**	**W12**	**W16**	**Sum**	**Average**
	Thr18	100	129	264	303	203	149	234	272	266	**1920**	**213**
	Ser21	99	143	218	250	216	166	238	285	271	**1886**	**210**
	Ser23	67	101	151	192	184	134	189	226	211	**1455**	**162**
	Ser24	16	27	39	55	44	39	46	68	69	**403**	**45**
	Ser25	26	36	38	65	45	37	46	72	69	**434**	**48**
	Ser28	124	157	298	348	236	181	274	308	301	**2227**	**247**
	Thr30	124	157	298	348	236	181	274	309	301	**2228**	**248**
	Thr207	573	735	755	808	505	679	593	656	668	**5972**	**664**
	Thr214	494	655	697	760	414	611	526	589	607	**5353**	**595**
	Ser225	493	618	712	788	346	647	559	609	635	**5407**	**601**
	**Sum**	**3212**	**4268**	**5193**	**5655**	**3484**	**4183**	**4162**	**5029**	**4903**		
	**Average**	**178**	**237**	**289**	**314**	**194**	**232**	**231**	**279**	**272**		

* The Indicated Sum and Average were calculated using on sites that showed evidence of the respective PTM. For phosphosites, only Thr18-Ser25 were considered; for *O*-glycosylation, only sites Thr207-Ser225 were considered. Serine (Ser), Threonine (Thr), weeks (W)

Donor two peptidome derived data											
**Peptidome Phosphorylation**	**Location**	**W1**	**W2**	**W3**	**W4**	**W6**	**W8**	**W10**	**W12**	**W16**	**Sum**	**Average**
	Thr18	107	52	59	13	79	62	44	28	10	**454**	**50**
	Ser21	508	368	390	209	454	382	339	210	208	**3068**	**341**
	Ser23	631	486	520	357	629	568	495	339	295	**4320**	**480**
	Ser24	769	695	797	743	973	932	945	676	751	**7281**	**809**
	Ser25	775	685	770	846	926	867	908	664	738	**7179**	**798**
	Ser28	8	6	6	12	7	21	6	12	6	**84**	**9**
	Thr30							1		1	**2**	**1**
	Thr207	3									**3**	**3**
	Thr214										**0**	**0**
	Ser225		1								**1**	**1**
	***Sum**	**2790**	**2286**	**2536**	**2168**	**3061**	**2811**	**2731**	**1917**	**2002**		
	***Average**	**2691**	**457**	**507**	**434**	**612**	**562**	**546**	**383**	**400**		
	**Location**	**W1**	**W2**	**W3**	**W4**	**W6**	**W8**	**W10**	**W12**	**W16**	**Sum**	**Average**
**Peptidome Glycosylation**	Thr18	5	5	3	7		3	5	7	4	**39**	**5**
	Ser21	6	10	6	8	1	3	8	11	6	**59**	**7**
	Ser23	6	9	6	8	1	3	9	11	9	**62**	**7**
	Ser24	1	7	3	6	3	4	7	6	6	**43**	**5**
	Ser25	1	4	4	4	3	2	7	3	7	**35**	**4**
	Ser28	2	4	6	7	6	5	10	3	8	**51**	**6**
	Thr30	2	5	4	5	6	5	10	3	6	**46**	**5**
	Thr207	144	151	43	29	57	45	40	11	26	**546**	**61**
	Thr214	184	177	58	44	87	61	54	21	37	**723**	**80**
	Ser225	13	17		6	13	9	3	1	2	**64**	**8**
	***Sum**	**341**	**345**	**101**	**79**	**157**	**115**	**97**	**33**	**65**		
	***Average**	**114**	**115**	**51**	**26**	**52**	**38**	**32**	**11**	**22**		
**Peptidome non-modified**	**Location**	**W1**	**W2**	**W3**	**W4**	**W6**	**W8**	**W10**	**W12**	**W16**	**Sum**	**Average**
	Thr18	655	692	776	1271	1044	1062	1145	925	1135	**8705**	**967**
	Ser21	308	436	512	1161	745	817	943	831	1002	**6755**	**751**
	Ser23	209	352	426	1062	639	688	859	739	969	**5943**	**660**
	Ser24	81	155	166	701	309	342	428	425	532	**3139**	**349**
	Ser25	78	175	206	617	373	419	483	457	565	**3373**	**375**
	Ser28	851	865	985	1483	1311	1274	1407	1124	1313	**10613**	**1179**
	Thr30	859	870	998	1500	1319	1296	1417	1139	1324	**10722**	**1191**
	Thr207	571	653	296	201	380	295	279	168	235	**3078**	**342**
	Thr214	553	652	315	217	378	298	291	177	255	**3136**	**348**
	Ser225	610	652	340	258	412	328	320	184	286	**3390**	**377**
	**Sum**	**7671**	**7903**	**6201**	**9508**	**8561**	**8020**	**8855**	**7030**	**8536**		
	**Average**	**426**	**439**	**345**	**528**	**476**	**446**	**492**	**391**	**474**		

* The Indicated Sum and Average were calculated using on sites that showed evidence of the respective PTM. For phosphosites, only Thr18-Ser25 were considered; for *O*-glycosylation, only sites Thr207-Ser225 were considered. Serine (Ser), Threonine (Thr), weeks (W)

Donor two proteome derived data											
	**Location**	**W1**	**W2**	**W3**	**W4**	**W6**	**W8**	**W10**	**W12**	**W16**	**Sum**	**Average**
**Proteome Phosphorylation**	Thr18	2								2	**4**	**2**
	Ser21	150	73	49	22	1	1	2	1	3	**302**	**34**
	Ser23	207	147	114	80	44	84	53	49	49	**827**	**92**
	Ser24	286	249	280	234	155	282	241	261	259	**2247**	**250**
	Ser25	282	247	273	229	155	283	240	267	251	**2227**	**247**
	Ser28			2	1				1		**4**	**1**
	Thr30										**0**	**0**
	Thr207	1	1	1		1	5				**9**	**2**
	Thr214		1								**1**	**1**
	Ser225			1							**1**	**1**
	***Sum**	**927**	**716**	**716**	**565**	**355**	**650**	**536**	**578**	**564**		
	***Average**	**925**	**179**	**179**	**141**	**89**	**163**	**134**	**145**	**113**		
	**Location**	**W1**	**W2**	**W3**	**W4**	**W6**	**W8**	**W10**	**W12**	**W16**	**Sum**	**Average**
**Proteome Glycosylation**	Thr18										**0**	**0**
	Ser21	1									**1**	**1**
	Ser23	1					1				**2**	**1**
	Ser24			1	1	2	2	1	1	1	**9**	**1**
	Ser25			1	1	2	2	1	1	1	**9**	**1**
	Ser28	1					2		1	1	**5**	**1**
	Thr30	1					2		1	1	**5**	**1**
	Thr207	44	77	64	50	55	66	36	34	39	**465**	**52**
	Thr214	74	100	98	78	82	97	57	53	70	**709**	**79**
	Ser225	13	20	17	11	18	10	14	13	10	**126**	**14**
	***Sum**	**131**	**197**	**179**	**139**	**155**	**173**	**107**	**100**	**119**		
	***Average**	**44**	**66**	**60**	**46**	**52**	**58**	**36**	**33**	**40**		
**Proteome non-modified**	**Location**	**W1**	**W2**	**W3**	**W4**	**W6**	**W8**	**W10**	**W12**	**W16**	**Sum**	**Average**
	Thr18	295	273	315	263	189	364	308	354	335	**2696**	**300**
	Ser21	157	221	284	263	208	382	324	375	360	**2574**	**286**
	Ser23	104	156	228	216	176	309	286	340	329	**2144**	**238**
	Ser24	27	56	64	63	66	114	101	130	121	**742**	**82**
	Ser25	32	59	73	70	68	113	105	125	132	**777**	**86**
	Ser28	318	309	349	304	229	400	350	392	384	**3035**	**337**
	Thr30	318	309	351	305	229	400	350	393	384	**3039**	**338**
	Thr207	435	503	514	476	445	612	299	390	421	**4095**	**455**
	Thr214	397	482	478	446	411	582	273	367	390	**3826**	**425**
	Ser225	402	480	504	463	429	588	295	378	411	**3950**	**439**
	**Sum**	**3379**	**4022**	**4348**	**4011**	**3529**	**5268**	**3700**	**4421**	**4414**		
	**Average**	**188**	**223**	**242**	**223**	**196**	**293**	**206**	**246**	**245**		

* The Indicated Sum and Average were calculated using on sites that showed evidence of the respective PTM. For phosphosites, only Thr18-Ser25 were considered; for *O*-glycosylation, only sites Thr207-Ser225 were considered. Serine (Ser), Threonine (Thr), weeks (W)

Supplemental Table S4: PSM percentage of phospho- and glycosite modifications by donor and time in the peptidome and proteome derived data

**Donor one peptidome**												
**Peptidome Phosphorylation**	**Location**	**W1**	**W2**	**W3**	**W4**	**W6**	**W8**	**W10**	**W12**	**W16**	**Sum**	**Average**
	Thr18	3	9	12	8	8	9	9	5	8	**72**	**8**
	Ser21	65	55	66	57	51	49	47	41	44	**476**	**53**
	Ser23	83	65	77	65	61	61	61	57	56	**587**	**65**
	Ser24	96	85	94	89	78	83	82	80	79	**767**	**85**
	Ser25	96	87	92	85	81	79	81	80	79	**760**	**84**
	Ser28		1			1		1			**4**	**0**
	Thr30										**0**	**0**
	Thr207	1	1			1	1				**5**	**1**
	Thr214		1								**1**	**0**
	Ser225										**0**	**0**
	***Sum**	**344**	**301**	**340**	**304**	**279**	**283**	**282**	**264**	**267**		
	***Average**	**69**	**60**	**68**	**61**	**56**	**57**	**56**	**53**	**53**		
	**Location**	**W1**	**W2**	**W3**	**W4**	**W6**	**W8**	**W10**	**W12**	**W16**	**Sum**	**Average**
**Peptidome Glycosylation**	Thr18										**1**	**0**
	Ser21			1		1	2		1	1	**7**	**1**
	Ser23		1	2		1	2	1	1	2	**9**	**1**
	Ser24		1	4	2	1		2	2	3	**15**	**2**
	Ser25			3			1	2	3	3	**12**	**1**
	Ser28	1						1		1	**4**	**0**
	Thr30	1						1	1	1	**4**	**0**
	Thr207	16	12	7	8	19	9	14	14	12	**111**	**12**
	Thr214	20	20	15	13	25	13	16	15	14	**150**	**17**
	Ser225	4	4	2	2	4	2	2	3	3	**25**	**3**
	***Sum**	**40**	**37**	**24**	**23**	**48**	**24**	**31**	**32**	**28**		
	***Average**	**13**	**12**	**8**	**8**	**16**	**8**	**10**	**11**	**9**		

* The Indicated Sum and Average were calculated using on sites that showed evidence of the respective PTM. For phosphosites, only Thr18-Ser25 were considered; for *O*-glycosylation, only sites Thr207-Ser225 were considered. Serine (Ser), Threonine (Thr), weeks (W)

**Donor one proteome derived data**												
	**Location**	**W1**	**W2**	**W3**	**W4**	**W6**	**W8**	**W10**	**W12**	**W16**	**Sum**	**Average**
**Proteome Phosphorylation**	Thr18				1						**1**	**0**
	Ser21	13	2	24	24	3		9	2	4	**81**	**9**
	Ser23	43	34	48	44	20	24	29	25	28	**295**	**33**
	Ser24	87	82	87	84	81	78	83	78	77	**735**	**82**
	Ser25	78	76	87	81	81	79	83	76	77	**718**	**80**
	Ser28										**0**	**0**
	Thr30										**0**	**0**
	Thr207		1		1			1			**3**	**0**
	Thr214										**0**	**0**
	Ser225										**1**	**0**
	***Sum**	**221**	**194**	**245**	**234**	**185**	**181**	**204**	**182**	**186**		
	***Average**	**44**	**39**	**49**	**47**	**37**	**36**	**41**	**36**	**37**		
	**Location**	**W1**	**W2**	**W3**	**W4**	**W6**	**W8**	**W10**	**W12**	**W16**	**Sum**	**Average**
**Proteome Glycosylation**	Thr18										**0**	**0**
	Ser21			1							**1**	**0**
	Ser23			1							**1**	**0**
	Ser24			5	4	2	3	2	3	1	**20**	**2**
	Ser25			3	3	2	3	2	3	1	**17**	**2**
	Ser28										**1**	**0**
	Thr30										**1**	**0**
	Thr207	9	8	10	13	13	12	14	14	14	**108**	**12**
	Thr214	17	14	14	16	18	19	19	17	19	**152**	**17**
	Ser225	2	3	2	3	4	3	2	3	4	**26**	**3**
	***Sum**	**27**	**24**	**26**	**32**	**34**	**35**	**36**	**34**	**37**		
	***Average**	**9**	**8**	**9**	**11**	**11**	**12**	**12**	**11**	**12**		

* The Indicated Sum and Average were calculated using on sites that showed evidence of the respective PTM. For phosphosites, only Thr18-Ser25 were considered; for *O*-glycosylation, only sites Thr207-Ser225 were considered. Serine (Ser), Threonine (Thr), weeks (W)

Donor two peptidome derived data											
	**Location**	**W1**	**W2**	**W3**	**W4**	**W6**	**W8**	**W10**	**W12**	**W16**	**Sum**	**Average**
**Peptidome Phosphorylation**	Thr18	14	7	7	1	7	6	4	3	1	**49**	**5**
	Ser21	62	46	43	15	38	32	26	20	17	**300**	**33**
	Ser23	75	58	55	25	50	45	37	31	23	**399**	**44**
	Ser24	90	82	83	51	76	73	69	61	59	**644**	**72**
	Ser25	91	80	79	58	71	67	65	59	57	**627**	**70**
	Ser28	1	1	1	1	1	2		1		**7**	**1**
	Thr30										**0**	**0**
	Thr207	1									**1**	**0**
	Thr214										**0**	**0**
	Ser225										**0**	**0**
	***Sum**	**333**	**272**	**267**	**151**	**242**	**223**	**201**	**175**	**157**		
	***Average**	**67**	**54**	**53**	**30**	**48**	**45**	**40**	**35**	**31**		
	**Location**	**W1**	**W2**	**W3**	**W4**	**W6**	**W8**	**W10**	**W12**	**W16**	**Sum**	**Average**
**Peptidome Glycosylation**	Thr18	1	1		1				1		**4**	**0**
	Ser21	2	2	1	1			1	1	1	**9**	**1**
	Ser23	3	2	1	1			1	1	1	**11**	**1**
	Ser24	1	4	2	1	1	1	2	1	1	**14**	**2**
	Ser25	1	2	2	1	1		1	1	1	**11**	**1**
	Ser28			1				1		1	**4**	**0**
	Thr30		1					1			**4**	**0**
	Thr207	20	19	13	13	13	13	13	6	10	**119**	**13**
	Thr214	25	21	16	17	19	17	16	11	13	**153**	**17**
	Ser225	2	3		2	3	3	1	1	1	**15**	**2**
	***Sum**	**47**	**43**	**28**	**32**	**35**	**33**	**29**	**17**	**23**		
	***Average**	**16**	**14**	**9**	**11**	**12**	**11**	**10**	**6**	**8**		

* The Indicated Sum and Average were calculated using on sites that showed evidence of the respective PTM. For phosphosites, only Thr18-Ser25 were considered; for *O*-glycosylation, only sites Thr207-Ser225 were considered. Serine (Ser), Threonine (Thr), weeks (W)

Donor two proteome derived data												
	**Location**	**W1**	**W2**	**W3**	**W4**	**W6**	**W8**	**W10**	**W12**	**W16**	**Sum**	**Average**
**Proteome Phosphorylation**	Thr18	1								1	**1**	**0**
	Ser21	49	25	15	8			1		1	**99**	**11**
	Ser23	67	49	33	27	20	21	16	13	13	**258**	**29**
	Ser24	91	82	81	79	70	71	70	67	68	**680**	**76**
	Ser25	90	81	79	77	70	71	70	68	66	**670**	**74**
	Ser28			1							**1**	**0**
	Thr30										**0**	**0**
	Thr207						1				**2**	**0**
	Thr214										**0**	**0**
	Ser225										**0**	**0**
	***Sum**	**297**	**236**	**208**	**190**	**160**	**164**	**156**	**148**	**148**		
	***Average**	**59**	**47**	**42**	**38**	**32**	**33**	**31**	**30**	**30**		
	**Location**	**W1**	**W2**	**W3**	**W4**	**W6**	**W8**	**W10**	**W12**	**W16**	**Sum**	**Average**
**Proteome Glycosylation**	Thr18										**0**	**0**
	Ser21	1									**1**	**0**
	Ser23	1									**1**	**0**
	Ser24			2	2	3	2	1	1	1	**10**	**1**
	Ser25			1	1	3	2	1	1	1	**10**	**1**
	Ser28										**1**	**0**
	Thr30										**1**	**0**
	Thr207	9	13	11	10	11	10	11	8	8	**91**	**10**
	Thr214	16	17	17	15	17	14	17	13	15	**141**	**16**
	Ser225	3	4	3	2	4	2	5	3	2	**29**	**3**
	***Sum**	**28**	**34**	**31**	**27**	**32**	**26**	**33**	**24**	**26**		
	***Average**	**9**	**11**	**10**	**9**	**11**	**9**	**11**	**8**	**9**		

* The Indicated Sum and Average were calculated using on sites that showed evidence of the respective PTM. For phosphosites, only Thr18-Ser25 were considered; for *O*-glycosylation, only sites Thr207-Ser225 were considered. Serine (Ser), Threonine (Thr), weeks (W)

Supplemental Table S5: Observed Skyline MS1 glycopeptide percentages of the ladder peptide from ($_{190}$AVPVQALLLNQ… to $_{200}$QELLLNPTHQIYPVTQPLAPVHNPISV$_{226}$) for donor one over lactation in the proteome and peptidome derived data

**Proteome derived data**									
	**Week 1**	**Week 2**	**Week 3**	**Week 4**	**Week 6**	**Week 8**	**Week 10**	**Week 12**	**Week 16**
$_{190}$AVPVQALLLNQELLLNPTHQIYPVTQPLAPVHNPISV$_{226}$									
AVPVQALLLNQELLLNPT[N_1H_1]HQIYPVTQPLAPVHNPISV	1	2	2	3	1	2	2	2	3
AVPVQALLLNQELLLNPT[N_1H_1]HQIYPVT[N_1H_1]QPLAPVHNPISV	1	1	2	4	1	3	3	3	3
$_{196}$LLLNQELLLNPTHQIYPVTQPLAPVHNPISV$_{226}$									
LLLNQELLLNPT[N_1H_1]HQIYPVTQPLAPVHNPISV	1	1	1	1	1	1	1	1	1
LLLNQELLLNPT[N_1H_1]HQIYPVT[N_1H_1]QPLAPVHNPISV	0	0	0	1	0	0	0	1	0
$_{197}$LLNQELLLNPTHQIYPVTQPLAPVHNPISV$_{226}$									
LLNQELLLNPT[N_1H_1]HQIYPVTQPLAPVHNPISV	1	1	1	1	1	1	1	1	1
LLNQELLLNPT[N_1H_1]HQIYPVT[N_1H_1]QPLAPVHNPISV	0	0	0	1	0	1	1	0	1

Weeks (W)

Peptidome derived data									
	Week 1	Week 2	Week 3	Week 4	Week 6	Week 8	Week 10	Week 12	Week 16
$_{190}$AVPVQALLLNQELLLNPTHQIYPVTQPLAPVHNPISV$_{226}$									
AVPVQALLLNQELLLNPT[N_1H_1]HQIYPVTQPLAPVHNPISV	3	3	3	2	2	1	1	1	1
AVPVQALLLNQELLLNPT[N_1H_1]HQIYPVT[N_1H_1]QPLAPVHNPISV	5	5	8	8	7	5	5	4	5
$_{195}$ALLLNQELLLNPTHQIYPVTQPLAPVHNPISV$_{226}$									
ALLLNQELLLNPT[N_1H_1]HQIYPVT[N_1H_1]QPLAPVHNPISV	5	3	4	12	8	5	3	6	5
$_{196}$LLLNQELLLNPTHQIYPVTQPLAPVHNPISV$_{226}$									
LLLNQELLLNPT[N_1H_1]HQIYPVTQPLAPVHNPISV	6	6	0	1	25	1	2	2	4
LLLNQELLLNPT[N_1H_1]HQIYPVT[N_1H_1]QPLAPVHNPISV	4	7	2	1	27	2	4	3	5
LLLNQELLLNPT[N_1H_1]HQIYPVT[$N_1H_1S_1$]QPLAPVHNPISV	10	11	0	0	9	1	1	1	1
$_{197}$LLNQELLLNPTHQIYPVTQPLAPVHNPISV$_{226}$									
LLNQELLLNPT[N_1H_1]HQIYPVTQPLAPVHNPISV	9	9	1	1	8	0	1	1	2
LLNQELLLNPT[$N_1H_1S_1$]HQIYPVTQPLAPVHNPISV	4	3	0	0	1	0	0	0	0
LLNQELLLNPT[N_1H_1]HQIYPVT[N_1H_1]QPLAPVHNPISV	10	11	2	2	16	1	2	3	3
LLNQELLLNPT[N_1H_1]HQIYPVT[$N_1H_1S_1$]QPLAPVHNPISV	8	8	1	0	2	0	0	0	0
$_{198}$LNQELLLNPTHQIYPVTQPLAPVHNPISV$_{226}$									
LNQELLLNPT[$N_1H_1S_1$]HQIYPVTQPLAPVHNPISV	1	1	0	0	0	0	0	0	0
LNQELLLNPT[N_1H_1]HQIYPVT[N_1H_1]QPLAPVHNPISV	4	7	1	1	7	1	1	1	2
LNQELLLNPT[$N_1H_1S_1$]HQIYPVT[N_1H_1]QPLAPVHNPISV	3	3	0	0	1	0	0	0	0
$_{199}$NQELLLNPTHQIYPVTQPLAPVHNPISV$_{226}$									

NQELLLNPT[N_1H_1]HQIYPVTQPLAPVHNPISV	1	2	0	0	2	0	0	1	1
$_{200}$QELLLNPTHQIYPVTQPLAPVHNPISV$_{226}$									
QELLLNPT[N_1]HQIYPVT[N_1]QPLAPVHNPISV	1	2	2	1	37	2	1	1	6

Weeks (W)

Supplemental Table S6: Comparative average glycosylation occupancy percentage data from Byonic PSM counts and Skyline MS1 integration of the peptidome and proteome data.

**Peptidome Glycosylation Data**									
	**W1**	**W2**	**W3**	**W4**	**W6**	**W8**	**W10**	**W12**	**W16**
**Donor one PSM counts**									
**Average percentage**	10	11	9	8	13	2	7	3	1
**Donor one MS1 intensities**									
**Average percentage**	5	5	2	2	10	1	1	2	2
**Proteome Glycosylation Data**									
	**W1**	**W2**	**W3**	**W4**	**W6**	**W8**	**W10**	**W12**	**W16**
**Donor one PSM counts**									
**Average percentage**	13	9	8	9	5	12	7	7	10
**Donor one MS1 intensities**									
**Average percentage**	5	5	7	11	4	8	7	8	9

* PSM count percentages and MS1 intensity percentages were curated from all possible PSMs from modified peptides with glycosylation at sites Thr207 or Thr214, from Supplemental Table 7. Data was matched by donor one, weeks 1-16, peptidome or proteome data, peptide sequences (ladder peptides from 190-226 to 200-226), glycan species (N_1, N_1H_1, $N_1H_1S_1$), and fragmentation type (EThcD). The only differences in the curation of the datasets was the score filtering, where PSM counts had Byonic Score ≥ 150, log probability ≥ 1.5 and Delta Mod Score > 5 and Skyline data had idot values ≥ 0.85.

Supplemental Table S7: Oxonium ions for mass spectrometry identification

**Oxonium ions**	**m/z**
Hex	127.0390
Hex	145.0495
Hex	163.0601
PHex	243.0264
PHex	405.0793
HexNAc	138.0550
HexNAc	168.0655
HexNAc	186.0761
HexNAc	204.0867
Neu5Ac	274.0921
Neu5Ac	292.1027
HexHexNAc	366.1395
HexNAcHexNAc	407.1660
HexHexNAcdHex	512.1974
HexHexNAcNeu5Ac	657.2349

Supplemental Table S8: Glycan species used for Byonic identifications

Glycan name	Short name	Chemical name	Mass (Da)
HexNAc(1)	N_1	$H_{13}C_8N_1O_5$	203.08
HexNAc(1)dHex(1)	N_1F_1	$H_{23}C_{14}N_1O_9$	349.14
HexNAc(1)Hex(1)	N_1H_1	$H_{23}C_{14}N_1O_{10}$	365.13
HexNAc(1)NeuAc(1)	N_1S_1	$H_{30}C_{19}N_2O_{13}$	494.17
HexNAc(1)Hex(1)dHex(1)	$N_1H_1F_1$	$H_{33}C_{20}N_1O_{14}$	511.19
HexNAc(1)Hex(1)NeuAc(1)	$N_1H_1S_1$	$H_{40}C_{25}N_2O_{18}$	656.23
HexNAc(1)Hex(1)dHex(2)	$N_1H_1F_2$	$H_{43}C_{26}N_1O_{18}$	657.25
HexNAc(1)Hex(1)dHex(1)NeuAc(1)	$N_1H_1F_1S_1$	$H_{50}C_{31}N_2O_{22}$	802.29
HexNAc(1)Hex(1)NeuAc(2)	$N_1H_1S_2$	$H_{57}C_{36}N_3O_{26}$	947.32

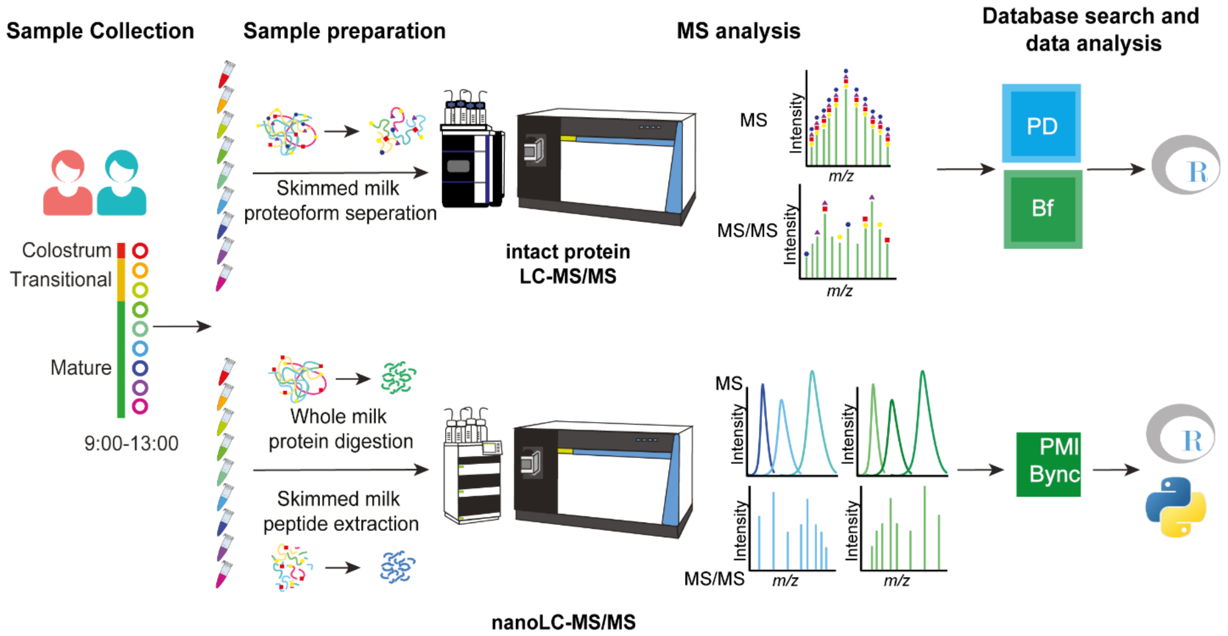

Supplemental Figure 1: Experimental scheme for personalized human milk PTM profiling. Samples from two individual donors were collected across lactation from weeks 1 to 16. Human milk proteoforms were first extracted and analyzed by intact protein LC-MS/MS, followed by protein and endogenous peptide extraction and analysis by label free quantification (LFQ) using LC-MS/MS in triplicate. All LFQ samples were ran as MS triplicates with varying MS/MS fragmentation types as higher-energy collisional dissociation (HCD), HCD-product-dependent stepping collision energy HCD (HCD-pd-sHCDs) and HCD-product-dependent electron-transfer/higher-energy collision dissociation (HCD-pd-EThcD). For intact proteoform analysis, isotopically resolved and unresolved spectra were deconvoluted with the BioPharma Finder software using Xtract or ReSpect algorithms respectively. For high-resolution proteoform MS/MS experiments, automatic searches were made in Thermo Proteome Discoverer software using ProSightPD nodes. All LFQ raw files obtained for the glycopeptide identification were processed in Byonic searching against a human β-casein protein database. For glycan analysis a database of 9 core 1 glycans was used. All data processing was done with Anaconda3 distribution of Python or R.

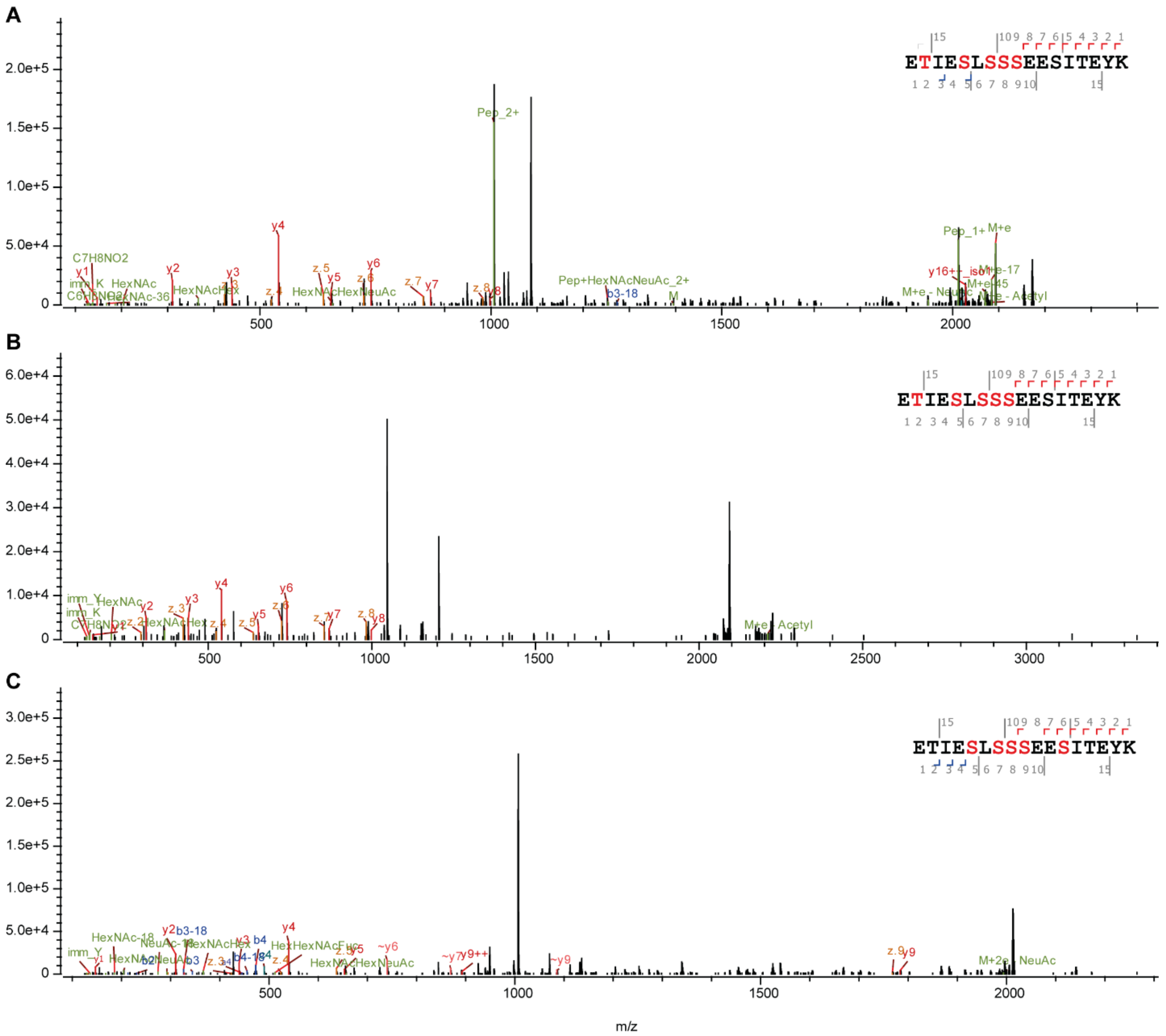

Supplemental Figure 2: EThcD fragmentation spectra of the N-terminal β-casein peptide $_{17}$ETIESLSSSEESITEYK$_{33}$ with negative O-glycan characteristics. All spectra are from donor one, week 16 peptidome data and passed through the strict filtering criteria. **A-C)** EThcD spectra with major abundant peaks unannotated, large indicated glycans on more than three sites (A) $N_1H_1S_2$, N_1S_1, N_1H_1, P, $N_1H_1F_2$ B) $N_1H_1S_2$ on 5 sites C) $N_1H_1S_2$, N_1F_1 on 4 sites) without corresponding oxomium ions and an absence of peptide fragments with retained phospho-/glyco- modifications.

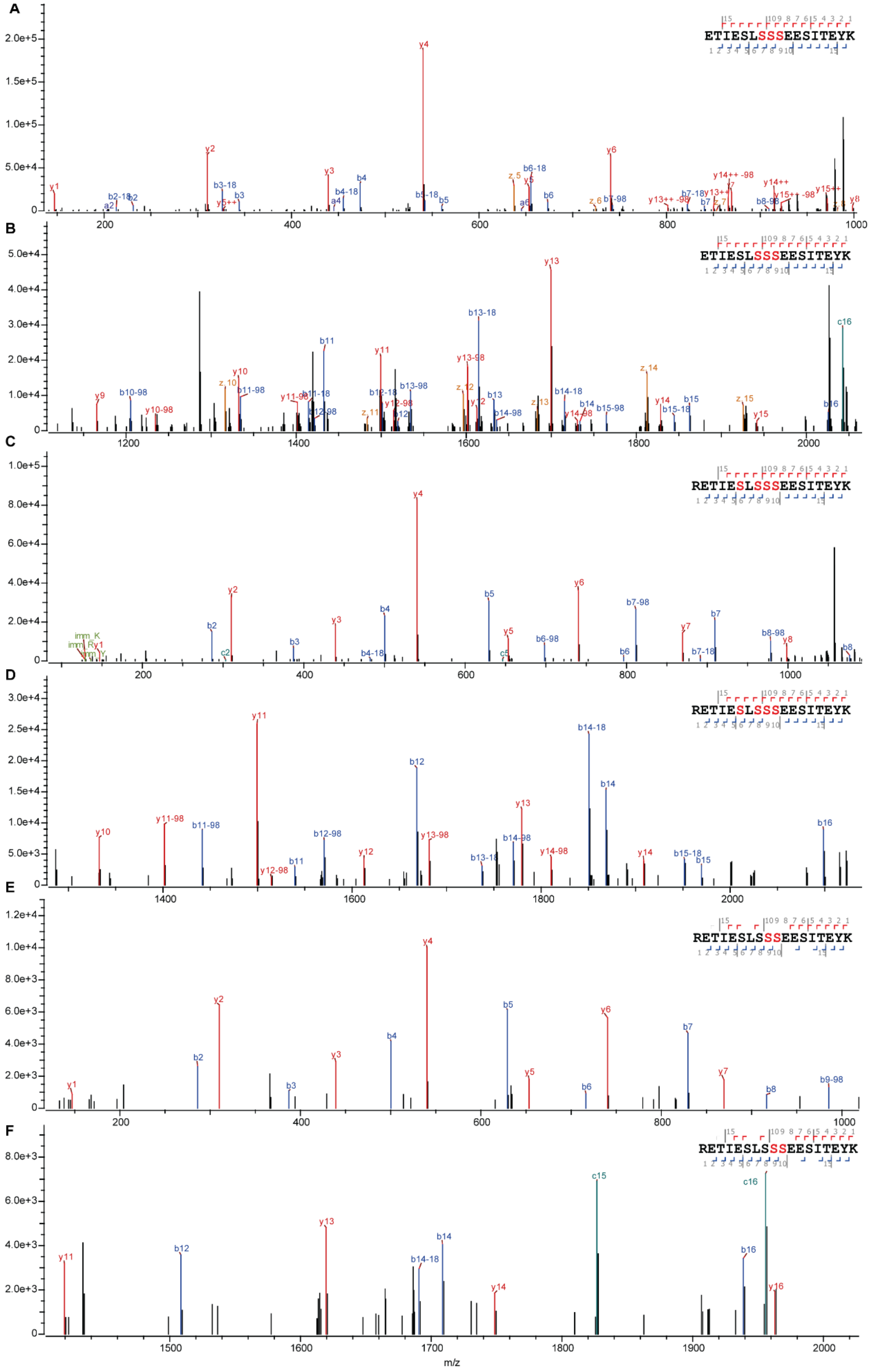
A
ETIESLSSSEESITEYK
B
ETIESLSSSEESITEYK
C
RETIESLSSSEESITEYK
D
RETIESLSSSEESITEYK
E
RETIESLSSSEESITEYK
F
RETIESLSSSEESITEYK
m/z

Supplemental Figure 3: Illustrative EThcD fragmentation spectra of β-casein phosphopeptides zoomed in regions. The N-terminal phospho peptides in figure 3 contain low intensity regions relative to the most intense ions, here these low intensity regions are displayed. **A)** Zoomed in region from *m/z* 100 to 1000 of the peptidome peptide with 3 phosphorylated Ser residues. **B)** Zoomed in region from *m/z* 1100 to 2000 of the peptidome peptide with 3 phosphorylated Ser residues. **C)** Zoomed in region from *m/z* 100 to 1000 of the peptidome peptide with 4 phosphorylated Ser residues. **D)** Zoomed in region from *m/z* 1300 to 2000 of the peptidome peptide with 4 phosphorylated Ser residues. **E)** Zoomed in region from *m/z* 100 to 1000 of the proteome peptide with 2 phosphorylated Ser residues. **F)** Zoomed in region from *m/z* 1400 to 2000 of the proteome peptide with 2 phosphorylated Ser residues. Designated phosphosites are indicated as red amino acids in the peptide sequence. Confidence in the phosphosite annotations are evident from the precursor mass with the neutral loss of phosphorylation from Ser (M−98 Da) upon fragmentation, with additional b- and y-ions from the peptide backbone placing the -98 Da at specific Ser residues.

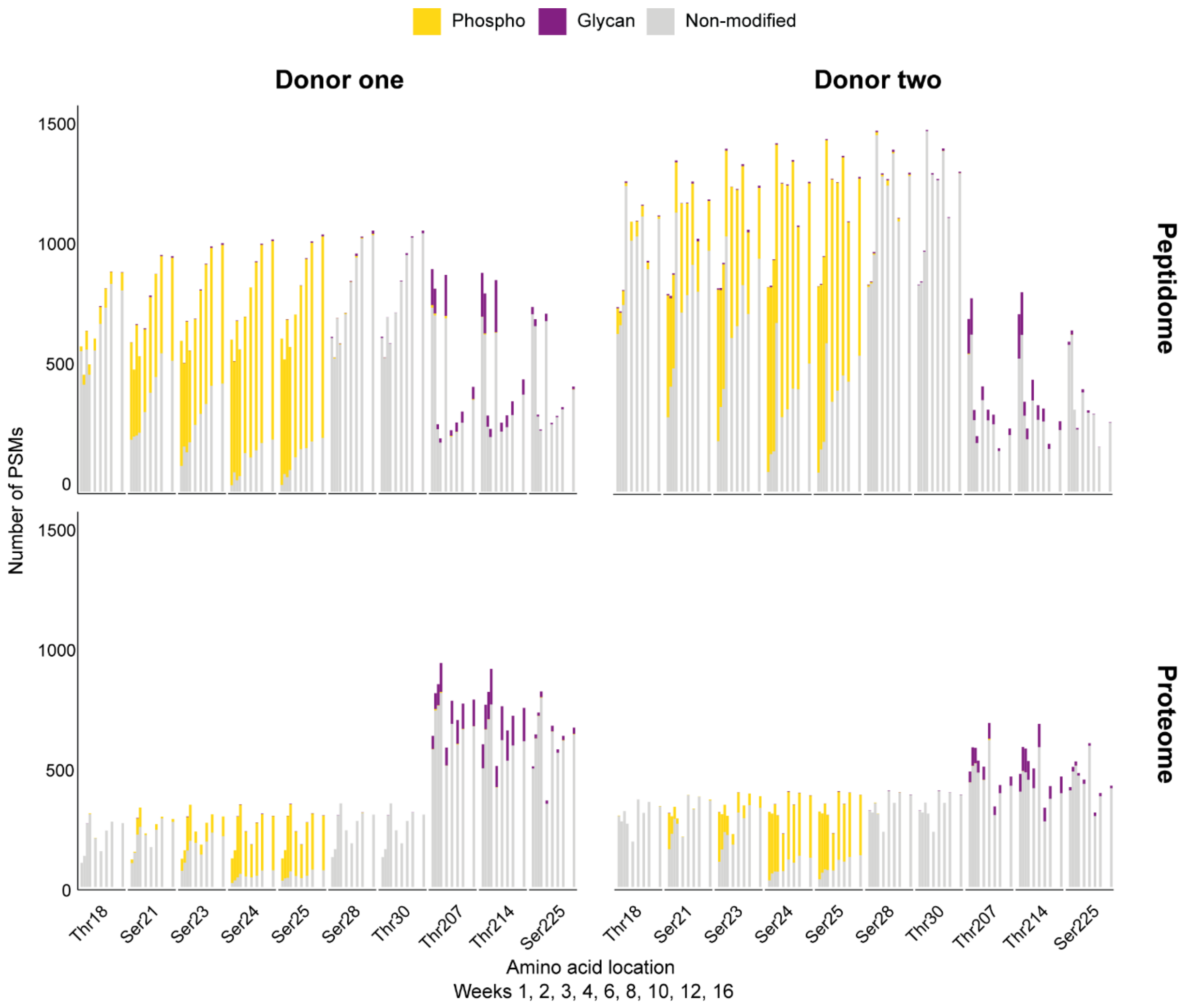

Supplemental Figure 4: PSM-based comparison of phosphorylated and glycosylated sites in the peptidome and proteome across lactation. The PTMs across lactation are displayed for the peptidome and proteome at the indicated Ser and Thr sites across the protein backbone for donors one (left) and two (right). PTMs are displayed as stacked bars of non-modified to modified PSMs per site across all points in lactation. Each stacked bar represents the number of identified PSMs for non-modified peptides (grey), modified phosphopeptides (yellow) and glycopeptides (purple). Donor specific and lactations trends indicate that PTMs are highly individual specific, though overall the peptidome displays a greater relative abundance of phosphorylated and glycosylated amino acids than the proteome.

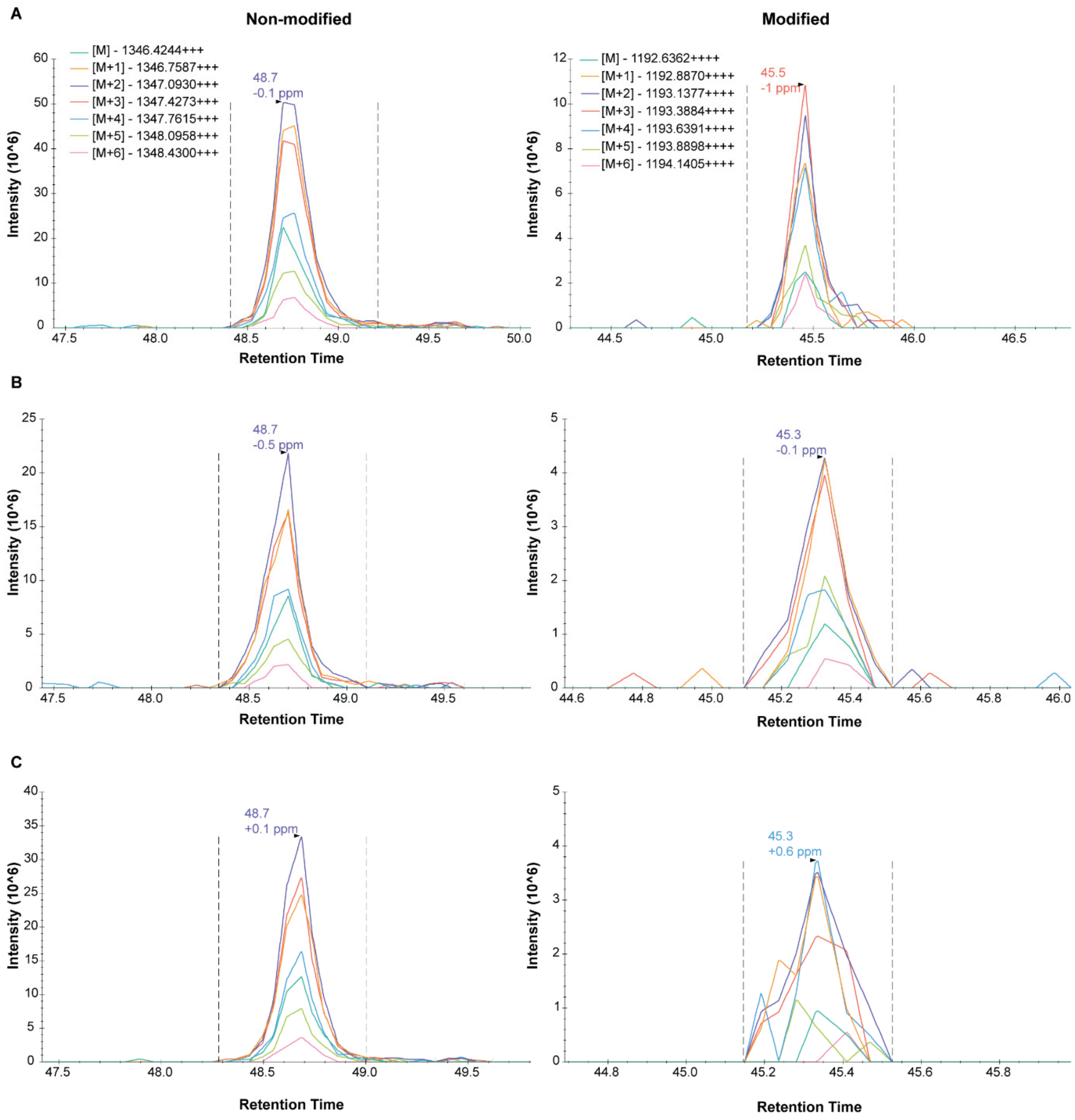

Supplemental Figure 5: MS1 ion traces of non-modified and modified C-terminal β-casein peptide $_{190}$AVPVQALLLNQELLLNPTHQIYPVTQPLAPVHNPISV$_{226}$ to validate glycan species quantification. All ion traces were extracted using Skyline software suite and are from donor one EThcD spectra across weeks 1, 6 and 16 of lactation for the non-modified (left) and glycosylated (right) peptide. The glycopeptide depicted at every time point has both sites Thr207 and Thr214 occupied with N_1H_1. The depicted ion traces show confident and reproducible integration of both the non-modified and glycosylated peptide with highly accurate retention times and low ppm error. **A)** The C-terminal β-casein peptide at week 1 of lactation. **B)** Peptide at week 6 of lactation **C)** Peptide at week 16 of lactation.

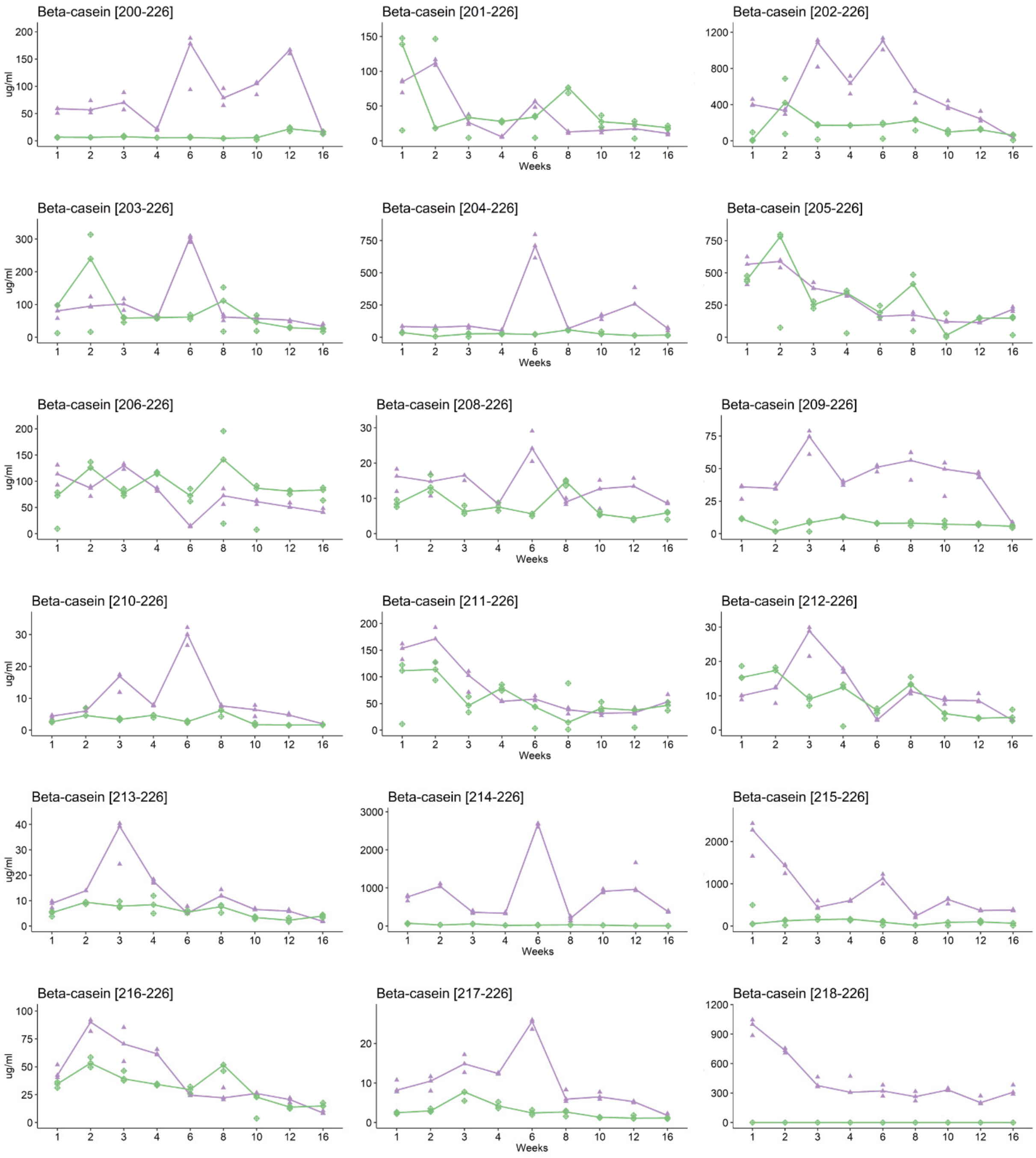

Supplemental Figure 6: Abundances in the peptidome of putative antimicrobial C-terminal β-casein peptides. Non-modified peptides were detected in both donors. Data for donor one and two are depicted in purple and green, respectively. Data points indicate the values of each technical replicate, lines are linked by the median of the data points in each week for each donor. The data show that every

ladder peptide has a different concentration in human milk and a distinctive longitudinal pattern. These ladder peptides may potentially also exhibit different functionality.
